# Intero-External Hemorrhoids Complicated by Ischio-Anal Infection Coexisting with Tubo-Ovarian Abscesses: A Case for Early Surgical Intervention

**DOI:** 10.7759/cureus.26117

**Published:** 2022-06-20

**Authors:** Julius M Nagaratnam, Astrid Manning, Lauren Hughes, Tarek Abi El Cheik, Frederick Tiesenga

**Affiliations:** 1 Surgery, Avalon University School of Medicine, Chicago, USA; 2 Surgery, Saint James School of Medicine, Chicago, USA; 3 Surgery, Washington University of Health and Science, Chicago, USA; 4 General Surgery, West Suburban Medical Center, Chicago, USA

**Keywords:** ischio-anal infection, deep pelvic abscess, conservative vs surgical management, hemorrhoidectomy, s: hemorrhoids

## Abstract

Hemorrhoids are abnormal collections of engorged blood vessels and tissue within the anal canal or surrounding the anus. Management consists of conservative treatment or a hemorrhoidectomy, dependent on disease severity, duration, and physician discretion. Reported is a case of a 44-year-old, African American female initially treated conservatively for intero-external hemorrhoids, that later abscessed into the ischio-anal fossa and was further complicated by an infection of the deep pelvic space. This report explores conservative and surgical management of hemorrhoids, and offers recommendations for symptom management, and reducing disease progression and complications.

## Introduction

A hemorrhoid is defined as an abnormal mass of dilated and engorged blood vessels in swollen tissue that can occur internally in the anal canal or externally around the anus. Hemorrhoids affect approximately 10 million people in the United States and may be considered one of the most common anorectal disorders in adults aged 45-65, often presenting with bleeding, pain, or itching [1]. When hemorrhoids occur internally, they often protrude through the outer sphincter of the anus, and when they occur externally, thrombosis and/or acute edema is of concern [2].

Hemorrhoids are further classified on whether they are internal or external, relative to the dentate line, but primarily divided by the two main venous plexuses affected, and then graded on the degree of prolapse. The American College of Gastroenterologists and the American Gastroenterological Association use a four-point grading system to assess the severity of hemorrhoids. Grade I occurs when hemorrhoid protrudes the anal canal but does not prolapse outside the anus; Grade II occurs when hemorrhoid protrudes the anus during straining or evacuation; grade III occurs when hemorrhoid protrudes the anus during straining or evacuation and must be manually returned; and grade IV remains prolapsed outside the anus [3].

Management for hemorrhoids consists of conservative treatment or hemorrhoidectomy surgery depending on the disease severity and duration. Conservative management is typically recommended as a preventative approach to alleviate pain and manage symptoms and consists of increasing fiber, rubber band ligation, over-the-counter medication treatments, regular exercise, and limited straining during defecation. Hemorrhoidectomy surgery is recommended for patients who exhibit severe symptoms, including bleeding, massive swelling, clots, acute/severe pain, and the presence of both internal and external hemorrhoids [4].

## Case presentation

A 44-year-old African American female with a past medical history significant for hypertension, asthma, Class I obesity (BMI = 31 kg/m^2^), and multiple external hemorrhoids, was admitted to the hospital on March 5, 2022 for bilateral lower abdominal pain, constipation, nausea, vomiting, dysuria, fever (38.3°C) and chills. Abdominal CT demonstrated findings suggestive of pelvic inflammatory disease which was managed with oral doxycycline. The patient also underwent clot removal from one of the hemorrhoids and was advised to continue conservative management with the use of topical hemorrhoid ointments and analgesics, and was discharged home.

The patient subsequently presented to the emergency department on March 9, 2022, complaining of persistent burning rectal pain occurring since her last hospital admission. She reported that the topical ointment and the use of oral narco-analgesics did not improve the pain, and she reported a new occurrence of non-bloody, non-purulent, foul-smelling, and clear discharge draining from one of the external hemorrhoids. The patient reported additional bilateral lower abdominal pain, nausea, and insomnia secondary to the pain. Labs on admission demonstrated severe leukocytosis with a white blood cell count of 23,500 and creatinine of 2.46, all other labs were within normal limits. The patient was tachycardic (104bpm), febrile (38.1°C), and hypertensive (143/85 mmHg). However, all other vitals were within normal limits. Physical examination demonstrated tenderness to palpation of the lower abdomen bilaterally. Digital examination demonstrated a grade IV thrombosed intero-external right anterior hemorrhoid, a grade IV thrombosed intero-external right posterior hemorrhoid, and a grade IV thrombosed intero-external left lateral hemorrhoid with non-bloody, non-purulent, foul-smelling active discharge which demonstrated concern for deep tissue infection. 

**Figure 1:**
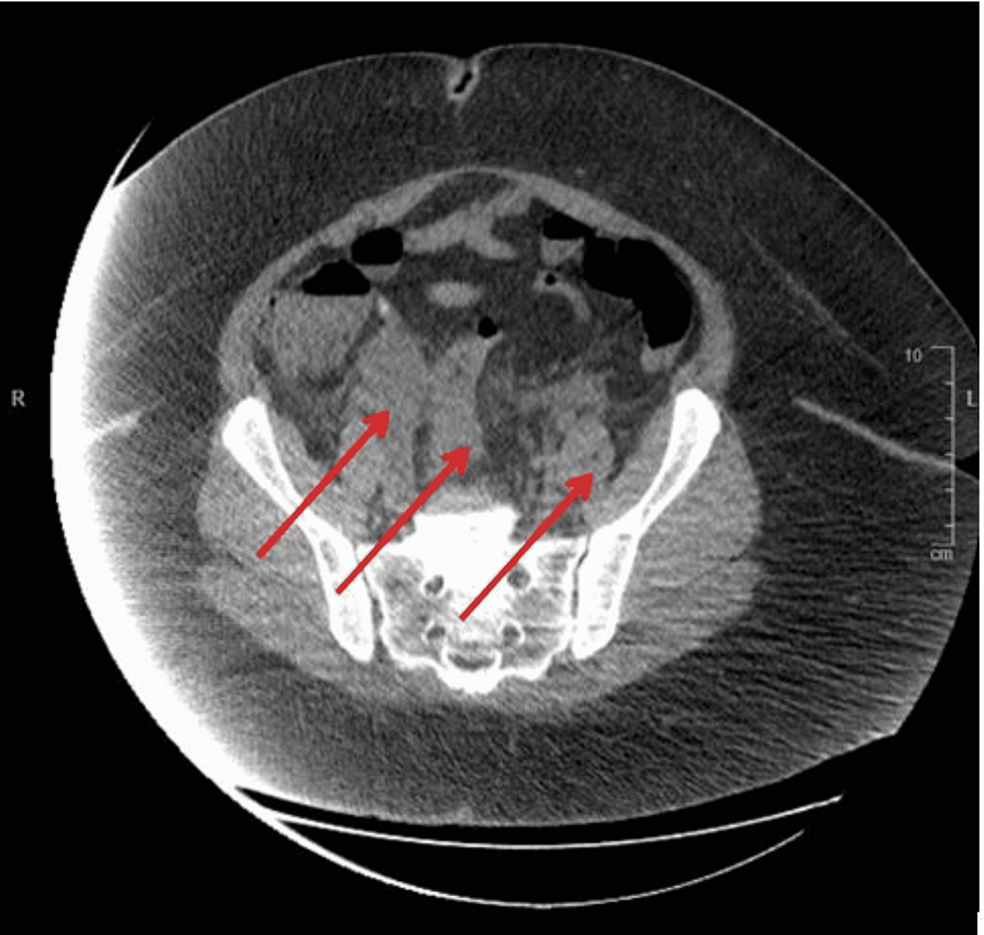
Abdominal CT showing undetermined pelvic fluid (indicated by red arrows)

An Abdominal CT (Figure 1) completed on admission demonstrated pelvic fluid with the possible differential diagnoses of a pyosalpinx, hydrosalpinx, tubo-ovarian abscess, or a cystic mass.

**Figure 2:**
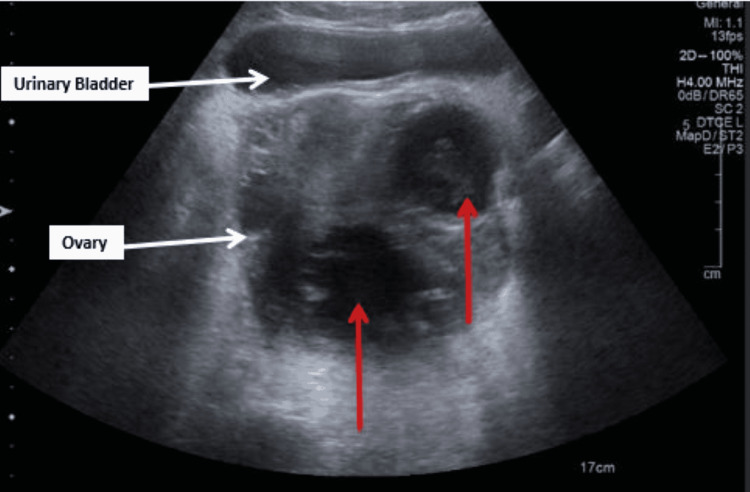
Transvaginal ultrasound image 1 showing 12cm by 6cm and 6cm by 4cm hypoechogenic lesions (indicated by red arrows)

**Figure 3:**
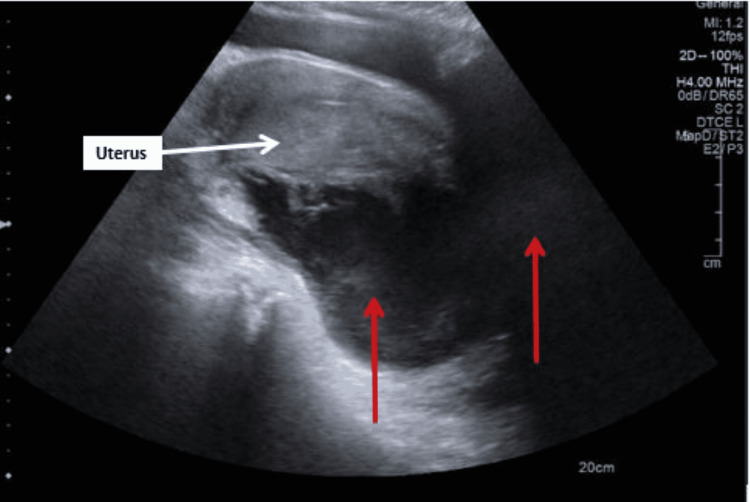
Transvaginal ultrasound image 2 showing 12cm by 6cm and 6cm by 4cm hypoechogenic lesions (indicated by red arrows)

Transvaginal ultrasound (Figures 2, 3) was also performed on admission and demonstrated a 12cm by 6cm and a 6cm by 4cm hypoechoic lesion in the pelvic space, suggesting tubo-ovarian abscesses.

The surgery team was consulted for the management of the hemorrhoids and the patient was scheduled for an urgent hemorrhoidectomy. Prior to the surgery, a preoperative checklist was performed, and written consent of the procedure was signed by the patient and witnessed by the attending nurse. The patient was taken to the surgery room, secured to the operation table in lithotomy position and general anesthesia was induced with the use of a laryngeal mask airway to maintain the airway. Perineal region prepped with povidone-iodine 7.5% scrub and appropriately draped.

A careful digital exam was first performed, followed by the insertion of an anal speculum. The left lateral hemorrhoid was approached, and perianal skin was grasped with Kocher's forceps. On compression of the surrounding skin, there was increased milky green foul-smelling drainage arising from hemorrhoid. The hemorrhoid was then elevated with a T-clamp, incised with LigaSure, further elevated off the sphincter mechanism, and then completely excised using a Whitehead extractor. The right anterior and right posterior hemorrhoids were excised using the same method. Chromic sutures and electrosurgical cautery tool were used to obtain hemostasis, and the region was irrigated with 0.9% saline. Gelfoam placed in the anal canal and dry dressing of 4x4 gauze and abdominal pads placed on the surgical region.

Pathology findings of the removed hemorrhoids demonstrated three dilated, congested, and thrombosed hemorrhoidal veins, with the left lateral hemorrhoid measuring 0.8cm by 0.8cm by 0.8cm, the right posterior hemorrhoid measuring 0.8cm by 1cm by 0.8cm and the right anterior hemorrhoid measuring 1cm by 1cm by 1cm, respectively.

Post-operatively, the patient was transferred to the inpatient wards. Infectious diseases placed the patient on intravenous doxycycline, ceftriaxone, and metronidazole, and performed a workup yielding negative blood cultures, RPR, and HIV screen (screening for Gonorrhea and Chlamydia was disclosed via outpatient). Obstetrics and Gynecology denied the necessity for surgical intervention for findings of pyosalpinx on transvaginal ultrasound and decided to manage her condition with oral antibiotics via outpatient. Nephrology was consulted for an elevated creatinine on admission and advised the use of conservative management and the avoidance of nephrotoxic medication. The patient’s white blood cell count decreased from 23,500 to 14,900 in the span of 24 hours, with no signs of sepsis. Signs and symptoms improved dramatically, and the patient denied acute complaints. Labs and vitals returned within normal limits. Surgery was signed off from the case on March 10, 2022, and the patient was discharged 24 hours later with a regimen of oral doxycycline and metronidazole.

## Discussion

The moment of occurrence of hemorrhoids is challenging to pinpoint since many patients are asymptomatic and seldom seek physician intervention. Although the exact cause of hemorrhoids is unknown, it arises due to various conditions, including strenuous bowel movements, diarrhea, obesity, anal intercourse, pregnancy, and pelvic dysfunction [5].

Conservative management

The initial conservative intervention that physicians recommend is increasing fiber intake as it softens stool and helps minimize bleeding and minor-to-temperate prolapses [6]. Ultraviolet coagulation and rubber band ligation are other forms of managing first and second-grade hemorrhoids, with ultraviolet coagulation preferred due to decreased pain and no mucopexy whereas rubber band ligation requires one to two banding sessions over a four-week period [1]. Over-the-counter medication treatments such as decongestants, nominal sedatives, protectants, astringents, topical nitroglycerin ointments, creams containing anti-inflammatories, antibiotics, native sedatives, and corticosteroids are useful for symptomatic management and rectal pain reduction [7,8]. Fitting sitz baths on toilet seats are another alternative for hemorrhoid-related pain management [4].

Conservative management is often the first treatment option; however, it is not curative and its efficacy is variable [4]. This case in particular was complicated by intero-external hemorrhoids that abscessed into the ischio-anal fossa, further complicated by a tubo-ovarian abscess in the deep pelvic space. This patient had a history of multiple intero-external hemorrhoids and conservative treatment with topical hemorrhoid ointments, analgesics, and other remedies failed. This brings to discussion if physicians should consider shorter trials of conservative management before transitioning to surgical treatment in order to alleviate patient pain and potential complications, which in this case was an abscess in the deep pelvic space. 

Surgical hemorrhoidectomy 

The primary objectives of hemorrhoidectomy surgery are to eliminate infectious hemorrhoidal strings and excessive tissue that instigates prolapsing, subsequent discomfort, and complications [9]. Surgeons determine the treatment intervention by measuring the prolapse scale and degree within the internal hemorrhoids. The Ferguson technique is the primary surgical intervention, in which a hill Ferguson retractor is inserted in the anal canal to evaluate the three hemorrhoidal strings prior to excision [1]. The Milligan-Morgan is the second main conventional procedure, which attempts to remove the hemorrhoids under general anesthesia, often with electrocautery, leaving raw patches in the anal canal and is less preferred due to increased postoperative pain [10]. Complications from a hemorrhoidectomy are rare but may include minimal tears, infection, peritonitis, urinary detainment, sphincter muscle damage, and anal stenosis [4].

Surgical hemorrhoidectomy is often the second treatment option for hemorrhoids as it is often reserved for severe symptoms like discharge, abscesses, clots, severe pain, and swollen external hemorrhoids as demonstrated in this case. Worsening progression in disease severity may have been avoided by the earlier surgical intervention [11,12]. Though surgical hemorrhoidectomy has risks of complication and infection the overall adverse risk is low and thus physicians should be undeterred in recommending this more aggressive management, especially in cases with complicating factors. As seen in the case, the patient’s pre-existing tubo-ovarian abscess and ischio-anal infection should have warranted a more aggressive approach [13,14] to avoid severe sepsis and other associated complications. Post-hemorrhoidectomy the patient reported resolution of symptoms and was able to be discharged home within 24 hours despite a complicated clinical presentation.

## Conclusions

Surgical intervention in the form of a hemorrhoidectomy is curative with a low overall adverse risk and thus should be more swiftly and readily considered. As in this case, had the patient received early surgical intervention for intero-external hemorrhoids, the future complication of ischio-anal infection could have been avoided. Henceforth this would have reduced the risk of the patient presenting with severe sepsis due to both infections of the deep pelvic space and ischio-anal fossa. Physicians should be mindful of the varying efficacy of conservative management and should consider more readily recommending a hemorrhoidectomy in the presence of multiple complicating factors.

## References

[1] Cristea C, Lewis CR (2022). Hemorrhoidectomy. https://www.ncbi.nlm.nih.gov/books/NBK549864/.

[2] Johanson JF, Sonnenberg A (1990). The prevalence of hemorrhoids and chronic constipation. Gastroenterology.

[3] Clinical Practice Committee, American Gastroenterological Association (2004). American Gastroenterological Association medical position statement: diagnosis and treatment of hemorrhoids. Gastroenterology.

[4] Sun Z, Migaly J (2016). Review of hemorrhoid disease: presentation and management. Clin Colon Rectal Surg.

[5] Lohsiriwat V (2015). Treatment of hemorrhoids: a coloproctologist's view. World J Gastroenterol.

[6] Garg P (2018). Conservative treatment of hemorrhoids deserves more attention in guidelines and clinical practice. Dis Colon Rectum.

[7] Mott T, Latimer K, Edwards C (2018). Hemorrhoids: diagnosis and treatment options. Am Fam Physician.

[8] Zagriadskiĭ EA, Bogomazov AM, Golovko EB (2018). Conservative treatment of hemorrhoids: results of an observational multicenter study. Adv Ther.

[9] Sanchez C, Chinn BT (2011). Hemorrhoids. Clin Colon Rectal Surg.

[10] Lu M, Shi GY, Wang GQ, Wu Y, Liu Y, Wen H (2013). Milligan-Morgan hemorrhoidectomy with anal cushion suspension and partial internal sphincter resection for circumferential mixed hemorrhoids. World J Gastroenterol.

[11] Sakr M (2014). Recent advances in the management of hemorrhoids. World J Surg Proced.

[12] Cerato MM, Cerato NL, Passos P, Treigue A, Damin DC (2014). Surgical treatment of hemorrhoids: a critical appraisal of the current options. Arq Bras Cir Dig.

[13] Moorthy K, Rao PP, Supe AN (2000). Necrotising perineal infection: a fatal outcome of ischiorectal fossa abscesses. J R Coll Surg Edinb.

[14] Zhang JF, Du ML, Sui HJ (2017). Investigation of the ischioanal fossa: application to abscess spread. Clin Anat.

